# Subacromial bursitis: current evidence and future directions in injection-based therapies–A narrative review

**DOI:** 10.1080/07853890.2026.2673146

**Published:** 2026-05-15

**Authors:** Jiaxing Ding, Sida Liu, Peilong Jiang, Huajun Jiang, Feng Han, Wei Qu

**Affiliations:** Department of orthopedics, The First Affiliated Hospital of Dalian Medical University, Dalian, China

**Keywords:** Subacromial bursitis, shoulder pain, corticosteroids, Hyaluronic acid, platelet-rich plasma, ultrasonography, interventional, review

## Abstract

**Objective:**

To review the anatomical and physiological characteristics of subacromial bursitis (SAB), clarify its pathogenesis and commonly used imaging diagnostic methods, and summarize the clinical efficacy, current application status, and future research directions of various injectable agents for SAB.

**Methods:**

This narrative review performed a non-systematic literature search in PubMed and Web of Science for injection therapies for SAB and related conditions , focusing on clinical trials and systematic reviews.

**Results:**

Corticosteroids (CS) remain the most widely used, providing robust short-term pain relief but with potential long-term complications. Randomized controlled trials suggest hyaluronic acid, nonsteroidal anti-inflammatory drugs, and hypertonic glucose offer alternative or adjunctive options. Emerging biologics like platelet-rich plasma (PRP) and interleukin-1 antagonists show promise but low-quality evidence. Ultrasound guidance enhances injection precision, and combining injection with physical rehabilitation optimizes outcomes.

**Conclusion:**

The choice of injectable agent requires balancing CS’s rapid effects against safer but less established alternatives. Future research should validate PRP, HA, IL-1Ra, and hypertonic glucose via large RCTs, develop combination strategies (e.g., CS+HA), integrate advanced ultrasound techniques (e.g., hydrodissection), and identify patient-specific factors (e.g., inflammatory endotypes, concomitant rotator cuff pathology) for personalized treatment.

## 1. Introduction

Shoulder pain is a prevalent and debilitating musculoskeletal condition that imposes a significant burden on global health [1]. The etiology of shoulder pain is diverse and often multifactorial. Common underlying conditions include rotator cuff pathologies (tendinopathy, partial or full-thickness tears), adhesive capsulitis, glenohumeral osteoarthritis, subacromial impingement syndrome(SIS), subacromial bursitis and instability. The subacromial bursa is the largest bursa in the human body and is essential for maintaining shoulder function, while also being a common site for inflammation. Repetitive friction and compression due to overuse, trauma, or impingement can lead to the development of subacromial bursitis (SAB) [1,2]. SAB is considered a subtype of subacromial impingement syndrome and is clinically defined as a painful shoulder disorder with distinct clinical and histopathological features [3]. It is widely recognized as a frequent cause of persistent shoulder pain, typically resulting in limitations in activities such as abduction and internal rotation, accompanied by functional impairment [1,3].

**Figure 1.**
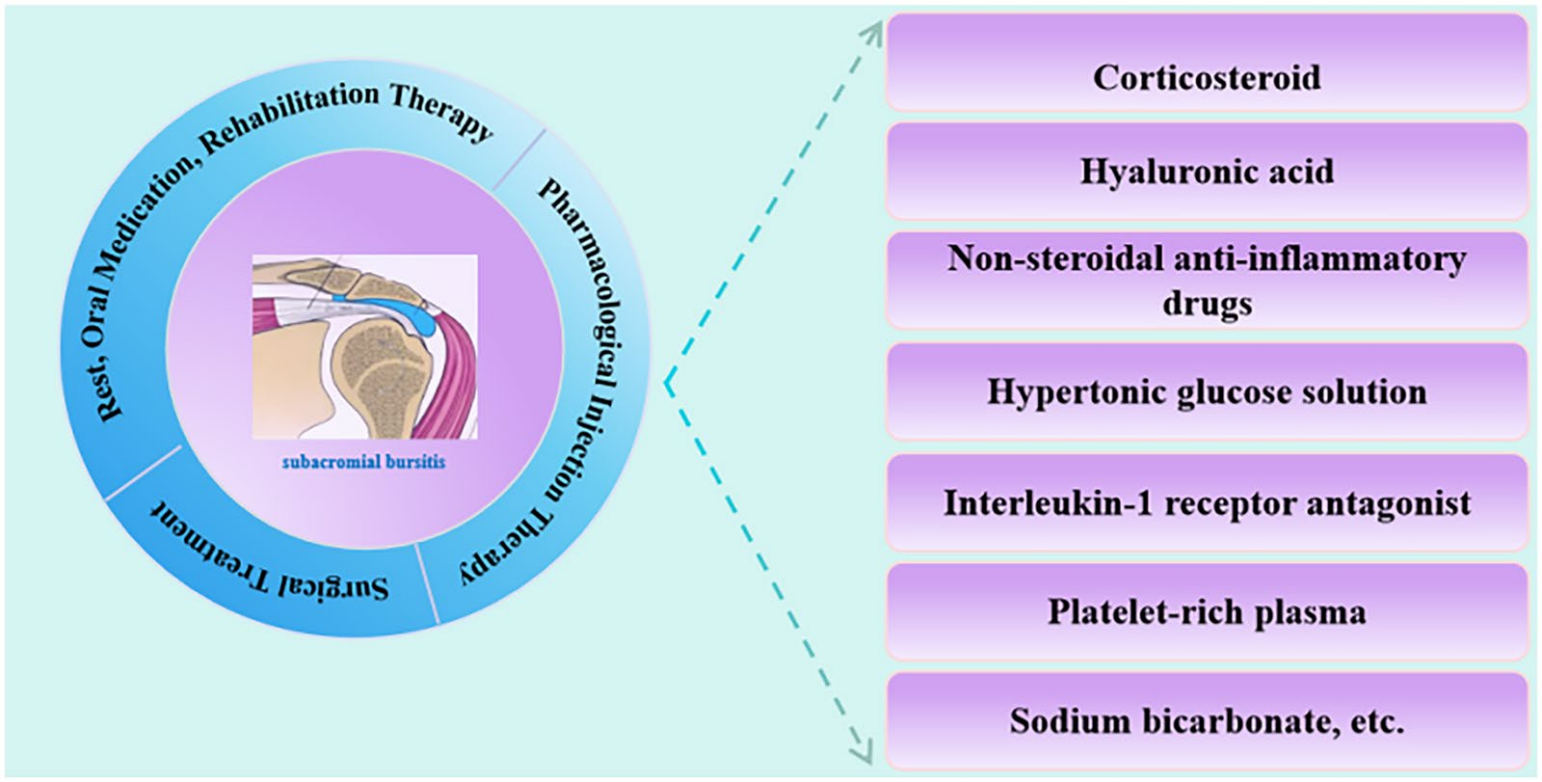
Treatment modalities for subacromial bursitis and medication options for injection therapy.

Current management strategies for SAB primarily involve non-surgical interventions, including rest, physical therapy, anti-inflammatory medications, and injection therapy. When prolonged conservative treatment yields insufficient results, arthroscopic surgical procedures such as bursectomy and acromioplasty may be considered to alleviate symptoms and improve function. Among non-surgical options, injection therapy is often regarded as a cornerstone of SAB management due to its minimal invasiveness, straightforward administration, favorable safety profile, and considerable therapeutic efficacy [1]. A variety of injectable agents are utilized clinically, with corticosteroids (CS) being the most conventional and widely used choice. Other agents include hyaluronic acid (HA), non-steroidal anti-inflammatory drugs (NSAIDs), hypertonic glucose solution (HGS), and interleukin-1 receptor antagonists (IL-1Ra), among others. However, debates persist regarding optimal agent selection, comparative effectiveness, ideal timing of injection, and associated adverse effects. Figure 1 illustrates the treatment approaches for subacromial bursitis and the medication options for injection therapy.

This review focuses on SAB. Nevertheless, SIS represents another disease entity that cannot be overlooked. It is noteworthy that SAB and SIS often coexist in clinical practice, but there are distinctions between the two conditions. SAB can be considered an early pathological change of SIS, specifically an inflammatory disease involving the subacromial bursa—the ‘cushioning pad’—characterized by aseptic inflammatory reactions such as hyperemia, edema, exudation, proliferation, thickening, and adhesion of the bursal wall [4–6]. In contrast, SIS is typically a syndrome resulting from narrowing of the subacromial space and increased pressure during shoulder movement, leading to repeated impingement between the greater tubercle of the humerus and the coracoacromial arch, thereby causing a cascade of pathological changes. This series of pathological alterations includes subacromial bursitis, rotator cuff tendinopathy (tendinitis/tendinosis), and partial or full-thickness rotator cuff tears [5,7]. However, regardless of whether the underlying etiology is SAB or SIS, the subacromial bursa itself is the direct target of injection therapy. Therefore, previous studies about injections for the treatment of SIS can provide a valuable reference for the present study. In the sections on NSAID and PRP injection therapies, this review cites numerous studies that investigated injection treatments for SIS, aiming to provide a more comprehensive reference for the treatment of SAB.

## 2. Anatomical and physiological characteristics of the subacromial bursa

The subacromial bursa, also known as the subdeltoid bursa, is a boat-shaped structure situated between the deep surface of the deltoid muscle, the acromion, the coracoacromial ligament, and the superficial surface of the rotator cuff tendons [8]. The center of the subacromial bursa lies at the level of the anterolateral angle of the acromion, extending mediolaterally and anteroposteriorly. Its roof originates from the anterolateral acromion and extends posteriorly to form the posterior shoulder curtain. The subacromial bursa frequently communicates with the subdeltoid bursa, with deltoid muscle fibers serving as lateral attachments. It possesses a relatively rich vascular network, receiving its blood supply from the acromial branch of the thoracoacromial artery, the anterior and posterior humeral circumflex arteries, the suprascapular artery, and direct branches from the axillary artery [9,10]. Innervation structures, including nerve fibers, free nerve endings, and other mechanoreceptors, are present within the bursal lining and sublining, indicating its potential role in nociception and proprioception [11].

Physiologically, the subacromial bursa functions as a cushion between the rotator cuff and adjacent structures. It is crucial for facilitating the gliding of the supraspinatus tendon within the subacromial space and reducing friction between the tendon and the coracoacromial arch during arm abduction [12]. Furthermore, recent studies have revealed that the bursa is rich in bioactive components such as stem cells, vascularization, and growth factors, with the stem cells possessing tenogenic differentiation potential [13]. This evidence suggests that the subacromial bursa may play an active role in the repair of rotator cuff tissue injuries [13].

The etiology of subacromial bursitis (SAB) is multifactorial. Common intrinsic and extrinsic factors include repetitive overuse, subacromial impingement, and acute trauma. Less frequent causes encompass the deposition of calcific material, infectious or immune-mediated systemic diseases, and even improper vaccination [14,15].

Histologically, the subacromial-subdeltoid (SASD) bursa is composed of two distinct layers: an inner intimal layer and an outer subintimal layer [16]. The intimal layer, which lines the bursal cavity, is typically 1–2 cells thick and contains two types of synoviocytes: macrophage-like type A cells, responsible for phagocytosis, and fibroblast-like type B cells, which produce hyaluronic acid and other components of the synovial fluid. Beneath this lies the subintimal layer, a loose connective tissue scaffold that houses the rich vascular and lymphatic network, as well as the neural elements (nerve fibers, free nerve endings, and mechanoreceptors) described previously. This subintimal layer also contains a heterogeneous population of cells, including fibroblasts, adipocytes, and the recently described progenitor cells with tenogenic potential [13,16]. Understanding this layered architecture is clinically relevant, as different injectable agents may primarily target different compartments. For instance, corticosteroids, with their potent anti-inflammatory effect, are thought to act on the cellular elements within both the intimal and subintimal layers. Hyaluronic acid may exert its visco-supplementive and anti-inflammatory effects on synoviocytes in the intimal layer. In contrast, biological agents like platelet-rich plasma (PRP) might aim to modulate the activity of the progenitor cells and fibroblasts within the subintimal layer to promote tissue regeneration and healing.

Furthermore, knowledge of the surrounding anatomical structures, including the deltoid muscle, rotator cuff tendons, and coracoacromial arch, is essential for guiding the accurate placement of the needle and minimizing the risk of iatrogenic injury [16]. Thus, a comprehensive appreciation of the local anatomy forms the foundation for safe and effective delivery of injectable therapies for subacromial bursitis.

## 3. Symptoms and signs of subacromial bursitis

The diagnosis of SAB requires a combination of patient history, physical examination, and imaging studies. Patients with SAB often present with varying degrees of shoulder pain and nocturnal pain, typically resulting from overuse and mechanical wear. Physical examination reveals tenderness over the subacromial space, which may radiate to the deltoid region. Some patients also exhibit signs of subacromial impingement syndrome, such as shoulder pain, apprehension, and weakness during overhead activities [17]. From a clinical perspective, certain features can help differentiate isolated SAB from other common shoulder disorders. Patients with SAB often report severe nocturnal pain, which may be more pronounced than in isolated rotator cuff tendinopathy, potentially due to the inflammatory nature of the bursa and its compression during sleep [18]. Furthermore, a key distinguishing feature from glenohumeral joint pathologies, such as adhesive capsulitis (frozen shoulder) or osteoarthritis, is that the passive range of motion (ROM) is typically preserved in isolated SAB [19]. While active movements, especially abduction and internal rotation, are painful and may be limited, the examiner is usually able to achieve a full or near-full passive ROM [20]. In contrast, adhesive capsulitis is characterized by a significant and often equal limitation of both active and passive motion [20].

Commonly employed physic al examination maneuvers include Neer’s impingement test, Hawkins-Kennedy test, painful arc sign, and empty can test [21]. Neer’s test is a reliable and sensitive examination for the diagnosis of subacromial impingement syndrome. In a prospective study involving 500 patients with shoulder pain, Desouza et al. found that the accuracy of Neer’s test in diagnosing subacromial bursitis reached 70.4% [22].

However, as shoulder pain symptoms often result from overlapping pathologies, further imaging is usually necessary for definitive diagnosis.

## 4. Imaging examination for subacromial bursitis

### 4.1. Musculoskeletal ultrasound

Musculoskeletal ultrasound is a dynamic, cost-effective, and highly sensitive tool for assessing SAB. A thorough understanding of the normal sonoanatomy of the SASD bursa is a prerequisite for accurate diagnosis and guided interventions. On ultrasound, the normal SASD bursa is often not distinctly visible as a separate structure but appears as a thin, flat, hypoechoic line (representing a small amount of physiological fluid and the intimal/subintimal layers) sandwiched between two hyperechoic layers. The deep hyperechoic line represents the interface with the rotator cuff tendons (primarily the supraspinatus), while the superficial hyperechoic layer corresponds to the peribursal fat tissue and the overlying deltoid muscle [23]

In SAB, this anatomy becomes distorted. Bursitis is typically defined by distension of the bursa with hypoechoic or anechoic fluid (exceeding 2 mm in thickness) and thickening of the bursal wall (the combined intimal and subintimal layers), which may appear hypoechoic due to edema or hyperechoic due to chronic fibrosis and synovial proliferation [24].

**Figure 2.**
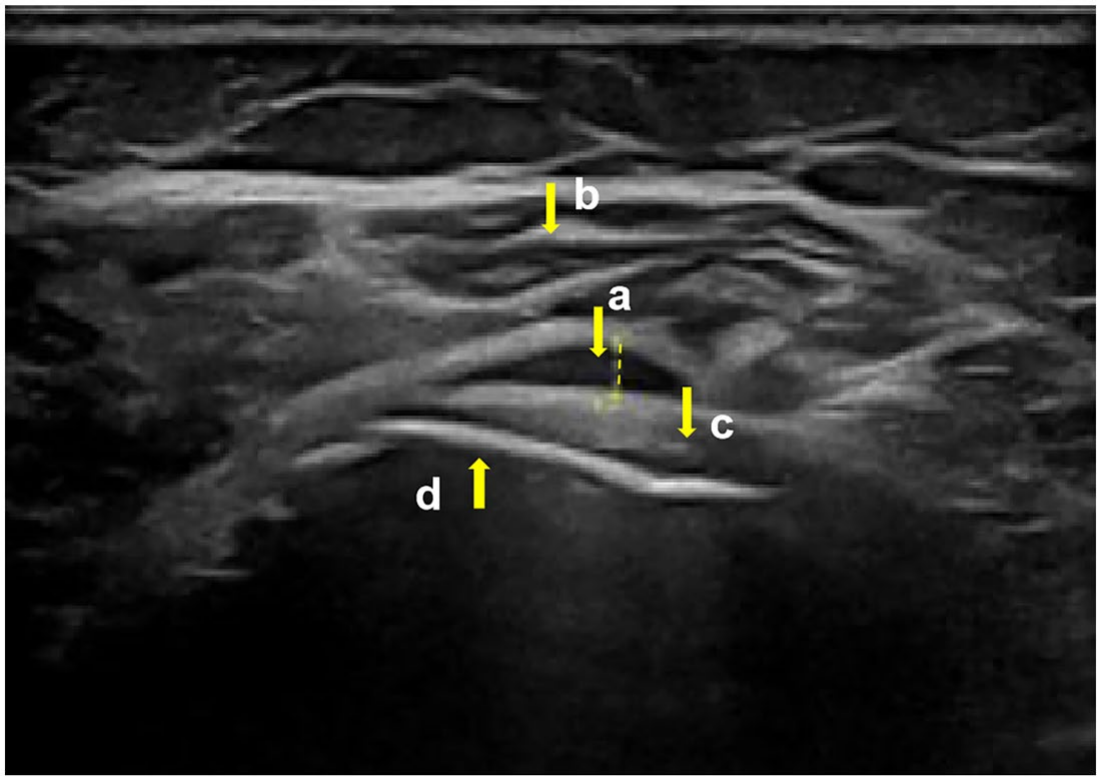
An ultrasound image of SAB. (a) Thickened and distended subacromial bursa containing anechoic fluid; (b) Deltoid muscle; (c) Humeral head; (d) Acromion.

This detailed sono-anatomical understanding is not just diagnostic but also guides therapeutic injections. The goal of a standard therapeutic injection is to place the needle tip within the hypoechoic bursal cavity to ensure the medication (e.g. corticosteroids, HA) bathes the inflamed intimal layer. However, other techniques exist. For instance, in patients with post-surgical or chronic adhesive bursitis, a hydrodissection technique can be employed. This involves the ultrasound-guided injection of a larger volume of fluid (e.g. normal saline, sometimes with anesthetic) into the hyperechoic peribursal fat plane to mechanically release adhesions between the bursal wall and surrounding tissues, thereby restoring normal gliding [23,25]. Recognizing these different tissue interfaces—the hypoechoic cavity versus the hyperechoic peribursal fat—is paramount for selecting and performing the appropriate interventional technique. Figure 2 is an ultrasound image of SAB.

### 4.2. Magnetic resonance imaging

MRI is also utilized to evaluate subacromial bursitis and associated pathologies, such as rotator cuff tendinopathy/tears, calcification of the coracoacromial ligament, and the presence of acromial spurs [26]. On MRI, SAB typically manifests as a distended subacromial-subdeltoid bursa containing fluid of variable signal intensity [10,27]. In acute bursitis, the bursal fluid appears hypointense on T1-weighted images and hyperintense on T2-weighted and proton density sequences. Synovial thickening and enhancement indicate active inflammation [27,28]. In chronic or fibrotic bursitis, the bursal wall may appear thickened and irregular, with loculated fluid collections [29]. Occasionally, rice bodies may be observed in longstanding inflammatory bursitis, appearing as intermediate-signal structures within the bursal fluid [10,27]. In calcific bursitis, calcific deposits appear as T2 hypointense, homogeneous ovoid signals, often accompanied by variable hyperintense peripheral signals representing concurrent edema or bursal fluid accumulation [28,30].

A recent arthroscopy-validated study demonstrated that MRI has a sensitivity of 97%, specificity of 63%, and accuracy of 92% for diagnosing subacromial-subdeltoid bursitis, with good interreader agreement (κ ≥ 0.68) [28]. However, it is important to note that the presence of subacromial bursitis on imaging does not reliably differentiate between symptomatic and asymptomatic shoulders, as a substantial proportion of asymptomatic individuals also exhibit these findings [30,31].

### 4.3. Others

The role of plain radiography should not be overlooked. Although it cannot directly visualize the subacromial bursa, it may provide diagnostic clues for subacromial bursitis by revealing subacromial osteophyte proliferation or calcifications, and can also aid in excluding other conditions such as calcific tendinitis.

## 5. Pharmacological injection therapy for subacromial bursitis

### 5.1. Corticosteroids

Corticosteroids (CS) are extensively used in orthopedics for various conditions, including adhesive capsulitis, osteoarthritis, tenosynovitis, and subacromial impingement [32,33]. Their therapeutic efficacy primarily stems from potent anti-inflammatory mechanisms and analgesic effects mediated through the inhibition of neuropeptides, such as substance P and calcitonin gene-related peptide [32]. In clinical practice, CS injections are the most widely utilized first-line pharmacologic treatment for SAB, demonstrating favorable clinical outcomes in alleviating pain and improving shoulder function [33]. Commonly employed CS agents include triamcinolone acetonide, methylprednisolone, hydrocortisone, and dexamethasone [34].

Multiple clinical controlled trials have shown that the short-term efficacy of other injectable agents, including hyaluronic acid, non-steroidal anti-inflammatory drugs (NSAIDs), and hypertonic glucose, is inferior to that of CS. However, there remains considerable variation in the reported optimal dosage and frequency of CS injections across studies [32]. Combining CS injection with rehabilitative exercise may yield additional benefits. A 2-year follow-up study by Zhu et al. [35] involving 64 SAB patients found that CS injection combined with progressive resistance training prolonged therapeutic efficacy and helped maintain long-term shoulder function. In a retrospective study evaluating dose-response effects, Yatish et al. [36] reported that a high-dose group (triamcinolone acetonide 20 mg) demonstrated superior outcomes compared to a low-dose group (triamcinolone acetonide 10 mg) at 12 weeks post-injection. They also noted that ultrasound-guided injection enhanced precision and minimized complications.

Concerns regarding potential complications often limit the clinical use of CS. These include transient hyperglycemia in diabetic patients and an increased risk of infection. CS injections can also adversely affect tendon metabolism and strength, potentially leading to tendon rupture, atrophy, and cutaneous atrophy [32–34]. Such effects may be associated with a higher failure rate following subsequent surgical rotator cuff repair. Other reported complications include detrimental effects on articular cartilage metabolism, septic arthritis, transient vasovagal reactions, post-injection pain flare, skin flushing, and cutaneous atrophy or hypopigmentation at the injection site [32].

Beyond complication risks, the long-term efficacy of CS remains questionable. Current evidence indicates that the peak benefit of CS injection is typically observed around the third week, after which symptoms may worsen, a pattern consistent with findings from earlier studies on subacromial impingement injections [35–37]. Similar concerns exist regarding CS use for rotator cuff injuries. Hopewell et al. [38] found that in the context of rotator cuff tears, the efficacy of CS injection peaks around week 8 and gradually diminishes over 6–12 months. While this finding cannot be directly extrapolated to isolated SAB, it highlights the potential uncertainty regarding the durability of efficacy following CS injection over time. Therefore, while CS demonstrates good short-term efficacy, its potential adverse effects cannot be overlooked. More high-quality research is needed to validate its long-term effectiveness, and a careful balance between therapeutic benefits and potential harms is essential before clinical application.

### 5.2. Hyaluronic acid

Hyaluronic acid (HA) is a ubiquitous component of the extracellular matrix, providing mechanical support, viscoelasticity, moisture retention, and anti-inflammatory effects to cells and tissues [39]. Clinical studies have confirmed that exogenous HA injections can alleviate pain, enhance function, and reduce friction between local tissues during the injury and repair processes of soft tissues such as bursae and tendons [40]. HA is widely used in sports medicine and is currently employed to treat various shoulder conditions, including subacromial impingement syndrome, adhesive capsulitis, and rotator cuff tears [41].

A meta-analysis of randomized controlled trials (RCTs) focusing on various shoulder disorders suggested that HA injections might be more effective than CS in treating chronic shoulder pain [42]. This may be attributed to HA’s ability to promote synoviocyte synthesis, inhibit inflammatory cell function, and block pain receptor signal transduction [42]. In an RCT comparing CS and HA for SAB, Hsieh et al. found that both agents were therapeutic. Although HA’s short-term efficacy was inferior to CS, it was deemed suitable for patients intolerant to CS [3]. Cheng et al. [43], in a 4-week follow-up study of 72 patients, reported that a combination of HA and compound betamethasone suspension provided more significant pain relief (2.08 ± 1.95 vs. 3.14 ± 2.0, *p* < 0.05) and greater improvement in abduction range of motion (7.12 ± 2.10 vs. 6.11 ± 1.93, *p* < 0.05) compared to betamethasone alone. Thus, in short-term follow-up, the combination yielded superior improvements in pain and active abduction function. HA is used not only in conservative management but also as a surgical adjunct. Animal studies have shown that HA injection can promote tendon-to-bone healing after rotator cuff repair in rabbits, increasing cartilage formation and collagen fiber maturity [44]. This indicates that HA may also exert beneficial effects on the rotator cuff.

Based on current clinical evidence, HA demonstrates acceptable short-term efficacy and can be considered for patients with contraindications to CS or used in combination with CS for injection therapy.

### 5.3. Non-steroidal anti-inflammatory drugs

Both Non-Steroidal Anti-Inflammatory Drugs (NSAIDs) and corticosteroids inhibit local inflammation. Given the known side effects associated with corticosteroid injections, some researchers propose that certain NSAIDs may serve as an alternative. Clinically, SAB and SIS are closely associated, and the subacromial bursa is frequently one of the core structures affected in SIS. Therefore, we have included studies on SIS patient populations in this section to provide important insights for the investigation of SAB treatment.

Min et al. [45] conducted an RCT involving 32 patients with subacromial impingement to compare the efficacy of triamcinolone acetonide and ketorolac. At the 4-week follow-up, ketorolac was associated with greater improvement in the UCLA shoulder score. Conversely, Goyal et al. [46], in a study of 70 patients, found no statistically significant differences in Visual Analogue Scale (VAS) and Shoulder Pain and Disability Index scores between ketorolac and methylprednisolone at the 12-week follow-up, a conclusion consistent with the findings of Kim et al. [47]. NSAIDs like lornoxicam or tenoxicam are considered weaker than ketorolac, and their clinical efficacy remains uncertain [46,48]. Some studies suggest that NSAID injections do not adversely affect cartilage, synovium, or chondrocyte viability. A systematic review and meta-analysis encompassing 10 studies concluded that subacromial NSAID injections provide comparable pain relief and functional improvement to CS injections in patients with impingement syndrome [33]. Theoretically, NSAID injections may be safer than CS injections in the long term [32].

### 5.4. Hypertonic glucose

Prolotherapy has been proposed as an alternative therapy for chronic rotator cuff tendinopathy [49]. Its mechanism primarily involves the precise injection of an irritant solution (typically a high-concentration glucose solution, sometimes mixed with a local anesthetic or other components) into damaged soft tissues such as ligaments, tendons, or joint capsules. This aims to induce a controlled, mild inflammatory reaction, thereby initiating and promoting the body’s intrinsic repair mechanisms to stimulate tissue regeneration and healing. Current research indicates that hypertonic glucose prolotherapy yields favorable clinical outcomes with a good safety profile in various musculoskeletal conditions, including lateral epicondylitis, plantar fasciitis, and rotator cuff tendinopathy [50].

In a double-blind RCT involving 54 SAB patients, Lin et al. [51] reported that a single 3 mL injection of 20% hypertonic glucose significantly reduced pain scores from 2 to 12 weeks post-injection, outperforming corticosteroid injections. However, at the 12-week mark, improvements in range of motion and overall function were greater in the CS group, indicating superior short-term efficacy for CS. Bursal thickness decreased in both groups over the 12-week follow-up period. In contrast, another study by Chang et al. [52] found that three injections of 15% hypertonic glucose yielded insignificant therapeutic benefits, providing insufficient evidence to support its clinical use for chronic shoulder pain and bursitis. Moreover, these injections were associated with increased tissue stiffness in the supraspinatus tendon. Currently, research on hypertonic glucose injections for SAB is limited. Further studies are required to identify appropriate patient subgroups and to determine the optimal concentration, volume, injection site, and frequency of glucose injections.

### 5.5. Interleukin-1 receptor antagonists

The bursal tissue of SAB patients exhibits increased levels of various inflammatory mediators, including cytokines such as SDF-1, IL-1β, and TNF-α, as well as pain-related mediators (cyclooxygenase-1 and cyclooxygenase-2) and proteolytic enzymes [8]. These play crucial roles in the development of inflammation and pain mediation. Consequently, therapies targeting specific inflammatory mediators have emerged as potential treatment options for SAB [8]. Earlier case reports suggested that injections of the IL-1 receptor antagonist anakinra could effectively alleviate symptoms [53]. However, anakinra has a short half-life, and its safety profile is not well-established. Subsequent research explored longer-acting IL-1 receptor antagonists, such as rilonacept. In an RCT involving 33 SAB patients randomized to receive either rilonacept or triamcinolone acetonide, Carroll et al. [54] found that rilonacept injections provided pain relief and functional improvement over a 4-week follow-up period, albeit with weaker efficacy than the corticosteroid group. The drug demonstrated mild and transient efficacy without serious adverse events. However, such biologic agents are costly, and more clinical research is warranted to confirm their efficacy, safety, and reliability.

### 5.6. Platelet-rich plasma

Platelet-rich plasma (PRP) is an autologous concentrate of platelets, leukocytes, and various growth factors derived from a patient’s own blood. It can stimulate cell proliferation, promote angiogenesis, and facilitate the regeneration of tendons, bone, and cartilage, while also reducing inflammation and alleviating pain. PRP is widely used in the treatment of rotator cuff injuries. Subacromial bursa-derived progenitor cells (SBDs) are recognized as an effective biological enhancer for promoting rotator cuff tendon healing. Relevant basic research has indicated that PRP possesses the potential to promote the proliferation of SBDs [55].

Clinically, SAB and SIS are closely associated, and SIS is characterized by a cascade of pathological changes. These pathological alterations include subacromial bursitis, rotator cuff tendinopathy (tendinitis/tendinosis), and partial or full-thickness rotator cuff tears [5]. SAB can exist either as a primary condition on its own or as one of the underlying causes of SIS, with the subacromial bursa frequently being one of the core structures affected in SIS [4,5]. Therefore, studies conducted in SIS populations can provide important insights for investigating the use of PRP in the treatment of SAB.

Šmíd et al. [56] administered three PRP injections and conducted a six-month follow-up, finding that at the final follow-up, the PRP group showed superior VAS and Constant scores compared to the corticosteroid-only group. Pasin et al. [57] reported similar findings. Their study included 90 patients with stage 2 SIS (concomitant SAB and rotator cuff tendinopathy) and divided them into three groups: PRP, corticosteroid, and exercise therapy. At the eight-week follow-up, the PRP group demonstrated statistically significant differences compared to the other two groups, with superior outcomes on the VAS, Disabilities of the Arm, Shoulder and Hand (DASH), and University of California Los Angeles (UCLA) scores. However, some studies have reported differing results. Barreto et al. [58] compared the efficacy of PRP and corticosteroids in SIS in a six-month follow-up study involving 40 patients (some of whom had SAB, with rotator cuff tears excluded). They found no significant differences between the two groups in DASH, UCLA, or Constant scores, suggesting no advantage for PRP injection. Additionally, Say et al. [56] conducted a randomized controlled trial involving 60 patients with SIS and found that at the six-week and six-month follow-ups, the corticosteroid group (a mixture of 40 mg methylprednisolone and 8 ml prilocaine) showed superior VAS pain scores and Constant scores compared to the PRP group, indicating a more pronounced efficacy for corticosteroids.

In summary, although no study has specifically confirmed the efficacy of PRP for SAB, its application in subacromial impingement suggests therapeutic potential for SAB. The efficacy of PRP is influenced by preparation methods, injection techniques, and individual variability. Regarding adverse effects, as PRP is derived from autologous blood, it carries a reduced risk of foreign body reactions and immune rejection. It has minimal side effects on the local tendon and intra-articular microenvironment, demonstrating a favorable safety profile and promising application prospects.

### 5.7. Other agents

Beyond the options above, other agents have been explored clinically. Studies indicate that local tissue acidosis in chronic bursitis adversely affects tissue and cellular functions, including impaired oxygen delivery, reduced energy production, and compromised glucose metabolism [59]. Based on this theoretical framework, Kang et al. [59] compared the efficacy of sodium bicarbonate injections with corticosteroid injections for SAB. In a 4-week follow-up study of 76 patients, they found the two treatments to be similarly effective.

Botulinum toxin has gained increasing use in treating musculoskeletal pain in recent years. Its analgesic mechanisms primarily include reducing muscle contraction, decreasing the release of local nociceptive mediators from autonomic and sensory fibers, stimulating endogenous enkephalin production, and inhibiting neurogenic inflammation and central sensitization. Lee et al. [60] found that at 1- and 3-month follow-ups, botulinum toxin type B injections resulted in better outcomes than corticosteroid injections on the Numeric Rating Scale (NRS), shoulder abduction angle, and Disability of the Arm, Shoulder, and Hand (DASH) questionnaire scores. They attributed this to the toxin’s high affinity for autonomic nerves, highlighting its therapeutic potential.

Normal saline can provide a certain degree of pain relief and improvement in shoulder range of motion. This effect may be attributable to the placebo effect, dilution of inflammatory mediators, the mechanical distention effect of the injection procedure, or the synergistic action of co-administered lidocaine. A recent study explored ultrasound-guided hydrodilatation for chronic SAB. The procedure involved injecting 0.9% normal saline into the bursa until fully distended, followed by a final injection of 2 ml of medium molecular weight hyaluronic acid and 40 mg of triamcinolone acetonide [25]. Results indicated that compared to triamcinolone injection alone, the hydrodilatation group achieved faster and better symptom relief without requiring re-treatment, suggesting superiority over traditional steroid injection [25]. Although these studies demonstrate promising short-term efficacy, further research is necessary to investigate their adverse effects, potential risks, and long-term outcomes.

## 6. Ultrasound-guided injection for the subacromial bursa: advantages and uncertaintie

Injection therapy is a conservative treatment modality characterized by its procedural simplicity. It can be employed as a standalone intervention for medication administration or combined with lavage techniques to remove inflammatory materials from the joint space. This approach facilitates direct contact between the therapeutic agent and the tissues within the joint capsule. By achieving targeted delivery, it circumvents ectopic effects and minimizes potential complications, thereby enabling the medication to exert its maximal therapeutic efficacy.

However, the effectiveness of ultrasound-guided treatment remains controversial. To explore whether there are differences between ultrasound-guided and landmark-guided corticosteroid injections into the subacromial bursa for treating subacromial bursitis and impingement syndrome, a systematic review found that in adult patients with subacromial bursitis, ultrasound-guided injections did not show a statistically significant difference in clinical improvement compared to landmark-guided injections [61]. However, another systematic review involving 12 randomized controlled studies suggested that ultrasound-guided corticosteroid injections might be superior to landmark-guided injections in improving clinical symptoms of subacromial impingement syndrome, although the study was limited by low-quality evidence [62]. The role of ultrasound-guided injections should not be overlooked. A recent study indicates that using ultrasound to guide saline injections to distend the bursa can achieve relatively good therapeutic outcomes [25].

Beyond its clinical advantages, the widespread adoption of ultrasound-guided injections is inherently linked to the operator’s skill and experience. The learning curve for mastering these procedures can be steep, particularly for beginners. Common pitfalls include poor needle-probe alignment, which can make the needle tip invisible; difficulties with hand-eye coordination; suboptimal needle entry planning that may lead to multiple skin punctures or inaccurate targeting; and challenges in handling long needles, which can easily deviate from the intended path [63]. Furthermore, distinguishing the true bursal cavity from the surrounding peribursal fat and ensuring the injectate is deposited within the target layer requires a nuanced understanding of sonoanatomy.

To overcome these initial challenges and enhance patient safety, structured training programs are essential. Basic and advanced phantoms, both commercial and homemade, have emerged as invaluable tools to accelerate the learning curve for medical students, residents, and practicing clinicians [63]. These phantoms allow trainees to practice needle-probe coordination, practice different injection techniques (e.g. in-plane vs. out-of-plane), and gain confidence in a risk-free environment before performing procedures on patients. Incorporating such simulation-based training into musculoskeletal curricula is highly recommended to optimize the reproducibility and success of ultrasound-guided bursal injections.

## 7. Surgical management of subacromial bursitis

Although the majority of patients with SAB achieve satisfactory outcomes following non-surgical management, surgical intervention becomes a critical option when conservative treatment fails. The primary goals of surgery are to address subacromial impingement, excise the inflamed pathological bursa, and manage the underlying etiology, thereby alleviating pain and restoring shoulder function [64].

Surgical treatment is typically indicated in the following scenarios: Patients whose symptoms persist or recur after at least 3–6 months of standardized, systematic non-surgical therapy—including pharmacological injections, physical therapy, and rehabilitation—with significant impact on daily activities and work [65]; Presence of definitive mechanical obstruction, such as structural stenosis caused by a large acromial spur or a hooked/curved acromion (Bigliani types II and III) [64,65]; Imaging-confirmed concomitant conditions like full-thickness rotator cuff tears, acromioclavicular joint pathology, or calcific tendinitis that are refractory to non-operative management; Severe limitation in shoulder range of motion due to extensive bursal hyperplasia and adhesions, particularly when differentiation from or co-existence with adhesive capsulitis is challenging [66].

A comprehensive evaluation and management of concomitant pathologies are imperative during surgery. If a rotator cuff tear is present, a repair should be performed based on the tear’s size and characteristics. For acromioclavicular joint with degenerative osteophytes or arthritis, a concomitant acromioclavicular joint resection may be indicated [66]. In cases of calcific tendinitis, the calcific deposit can be localized and removed under arthroscopic guidance [65]. Postoperative rehabilitation is as crucial as the surgical procedure itself to active movements and strength training, aiming ultimately for the restoration of comprehensive shoulder function [64,67]. Table 1 summarizes the mechanisms of action, advantages, and disadvantages of each injectable agent.

**Table 1.** Summary of advantages and disadvantages of common injectable agents for subacromial bursitis.

Agent	Mechanism of Action	Advantages	Disadvantages
CS	Potent anti-inflammatory, inhibition of neuropeptides	Rapid, effective short-term pain relief; gold standard for comparison	Potential tendon damage/rupture, hyperglycemia, skin atrophy, questionable long-term efficacy
HA	Visco-supplementation, anti-inflammatory, promotes cell synthesis	Good safety profile, suitable for CS-intolerant patients, potential for tendon healing	Slower onset, efficacy may be inferior to CS in short-term
NSAIDs	Inhibition of local inflammation	Avoids steroid-specific side effects (e.g., tendon damage), comparable efficacy to CS in some studies	Weaker anti-inflammatory effect than some CS, optimal agent unclear
Hypertonic Glucose	Induces controlled mild inflammation to stimulate repair	Potential for longer-term tissue regeneration, good safety profile	Inconsistent evidence, may increase tissue stiffness, optimal protocol unclear
IL-1Ra	Targeted inhibition of key inflammatory cytokine (IL-1)	Targeted therapy addressing specific inflammatory pathway	High cost, weak short-term efficacy compared to CS, limited safety data
PRP	Releases growth factors to stimulate regeneration and modulate inflammation	Autologous, good safety profile, promotes healing potential, favors long-term recovery	Efficacy varies by preparation, no SAB-specific RCTs
Other Agents	Reduces muscle contraction, inhibits nociceptive mediators	Alternative mechanism for refractory pain	Limited evidence, potential muscle weakness

CS: Corticosteroids; HA: Hyaluronic Acid; NSAIDs: Non-Steroidal Anti-Inflammatory Drugs; IL-1 Ra: Interleukin-1 Receptor Antagonists; PRP: Platelet-Rich Plasma; Other Agents: Sodium bicarbonate, Botulinum toxin and Normal saline.

## 8. Summary and future perspectives

SAB is a prevalent cause of shoulder pain, often coexisting with SIS but possessing distinct pathological features. A comprehensive understanding of its layered histological architecture—particularly the intimal layer, which is the primary target for anti-inflammatory agents, and the subintimal layer, which harbors progenitor cells responsive to biological agents—is fundamental to optimizing injection therapy.

CS injections remain the most established first-line intervention, offering rapid and effective short-term pain relief. However, their well-documented risks, including tendon damage, hyperglycemia, and questionable long-term durability, necessitate careful patient selection and consideration of alternatives. HA provides a safer profile and may serve as an alternative or adjunct to CS, particularly in patients with contraindications to steroids. NSAID injections, especially ketorolac, have shown comparable efficacy to CS in SIS populations, though direct evidence in isolated SAB remains limited. Hypertonic glucose prolotherapy demonstrates potential for longer-term tissue regeneration but is hampered by inconsistent evidence and unresolved questions regarding optimal protocols.

Emerging biological agents, particularly PRP, hold promise based on their regenerative and anti-inflammatory properties. However, the field faces a critical evidence gap: no study has specifically evaluated PRP for isolated SAB, and the efficacy of PRP is highly dependent on preparation method (leukocyte-rich vs. leukocyte-poor). Given that the subacromial bursa is a core structure affected in both SAB and SIS, well-designed studies in SIS populations with confirmed bursal involvement can provide valuable preliminary insights. Future investigations should prioritize SAB-specific randomized controlled trials that rigorously characterize PRP composition and directly compare different formulations.

Ultrasound guidance has emerged as a valuable tool for enhancing injection accuracy and enabling advanced techniques such as hydrodissection for chronic adhesive bursitis. However, the widespread adoption of this technique requires structured training programs, including the use of phantoms, to overcome the steep learning curve and ensure consistent, safe application.

In summary, the management of SAB is evolving from a one-size-fits-all approach toward more precise, mechanism-based strategies. Key priorities for future research include: (1) conducting large, multicenter randomized controlled trials that directly compare emerging agents (e.g. PRP, HA) against CS in well-phenotyped SAB populations; (2) validating combination therapies, such as CS with HA, to achieve synergistic effects; (3) exploring the role of advanced ultrasound-guided interventions, including hydrodissection, for refractory cases; and (4) identifying biomarkers or imaging characteristics that predict individual patient responses to specific injectable agents. By addressing these priorities, clinicians will be better equipped to deliver personalized, effective, and safe care for patients suffering from subacromial bursitis.

## Abbreviations

A list of abbreviations is given in Table 2.

**Table 2.** List of abbreviations.

Abbreviation	Full term
SAB	Subacromial Bursitis
CS	Corticosteroids
HA	Hyaluronic Acid
NSAIDs	Non-Steroidal Anti-Inflammatory Drugs
PRP	Platelet-Rich Plasma
IL-1Ra	Interleukin-1 Receptor Antagonists
MRI	Magnetic Resonance Imaging
VAS	Visual Analogue Scale
SBDs	Subacromial Bursa-derived Progenitor Cells

## Data Availability

Data availability is not applicable to this article as no new data were created or analysed in this study.

## References

[1] Lanham NS, Swindell HW, Levine WN. The subacromial bursa: current concepts review. JBJS Rev. 2021;9(11):110. doi: 10.2106/jbjs.Rvw.21.00110. 34757977

[2] Zoshima T. Clinical Images: subacromial bursitis with rice bodies as an initial solo presentation of rheumatoid arthritis. Arthritis Rheumatol. 2025;77(10):1451–1451. doi: 10.1002/art.43189. 40254915

[3] Hsieh LF, Lin YJ, Hsu WC, et al. Comparison of the corticosteroid injection and hyaluronate in the treatment of chronic subacromial bursitis: a randomized controlled trial. Clin Rehabil. 2021;35(9):1305–1316. doi: 10.1177/02692155211007799. 33858205

[4] Bhatti H, Operti ND, Jildeh TR. Metabolic functions of the subacromial bursa: implications for shoulder health and pathology. JBJS Rev. 2025;13(12):189. e25.00189 doi: 10.2106/jbjs.Rvw.25.00189. 41401261

[5] Al Hammadi MI, Shah ZA, Rathod RK, et al. Shoulder impingement pain syndrome: pathophysiology, diagnosis, and a review of current treatment strategies. Cureus. 2025;17(9):e92045. doi: 10.7759/cureus.92045. 41080250 PMC12514857

[6] Hanchard NC, Lenza M, Handoll HH, et al. Physical tests for shoulder impingements and local lesions of bursa, tendon or labrum that may accompany impingement. Cochrane Database Syst Rev. 2013;2013(4): cd007427. doi: 10.1002/14651858.CD007427.pub2. 23633343 PMC6464770

[7] Witten A, Clausen MB, Thorborg K, et al. Bilateral ultrasonographic findings in patients with unilateral subacromial pain syndrome and intact rotator cuff tendons. J Shoulder Elbow Surg. 2025;34(11):e1017–e1025. doi: 10.1016/j.jse.2025.02.020. 40089008

[8] Klatte-Schulz F, Thiele K, Scheibel M, et al. Subacromial bursa: a neglected tissue is gaining more and more attention in clinical and experimental research. Cells. 2022;11(4):663. doi: 10.3390/cells11040663. 35203311 PMC8870132

[9] Põldoja E, Rahu M, Kask K, et al. Blood supply of the subacromial bursa and rotator cuff tendons on the bursal side. Knee Surg Sports Traumatol Arthrosc. 2017;25(7):2041–2046. doi: 10.1007/s00167-016-4379-4. 27872990

[10] Nakai D, Fukuta S, Kawamata J, et al. Pathological formation of subcoracoid bursa effusion on magnetic resonance imaging studies. J Shoulder Elbow Surg. 2025;34(6):e340–e347. doi: 10.1016/j.jse.2024.09.033. 39603382

[11] Kennedy MS, Nicholson HD, Woodley SJ. The morphology of the subacromial and related shoulder bursae. An anatomical and histological study. J Anat. 2022;240(5):941–958. doi: 10.1111/joa.13603. 34865216 PMC9005683

[12] Marshall BP, Ashinsky BG, Ferrer XE, et al. The subacromial bursa modulates tendon healing after rotator cuff injury in rats. Sci Transl Med. 2024;16(744):eadd8273. doi: 10.1126/scitranslmed.add8273. 38657023 PMC11646107

[13] Morikawa D, Johnson JD, Kia C, et al. Examining the potency of subacromial bursal cells as a potential augmentation for rotator cuff healing: an in vitro study. Arthroscopy. 2019;35(11):2978–2988. doi: 10.1016/j.arthro.2019.05.024. 31629585

[14] Chuaychoosakoon C, Boonsri P. Partial infraspinatus tear with bursitis following an mRNA vaccination: a case report. Ann Med Surg (Lond). 2023;85(5):2159–2161. doi: 10.1097/ms9.0000000000000655. 37229000 PMC10205253

[15] Reitsema RD, Jiemy WF, Wekema L, et al. Contribution of pathogenic T helper 1 and 17 cells to bursitis and tenosynovitis in polymyalgia rheumatica. Front Immunol. 2022;13:943574. doi: 10.3389/fimmu.2022.943574. 36032100 PMC9402989

[16] Ricci V, Ricci C, Tamborrini G, et al. From histology to sonography in synovitis: EURO-MUSCULUS/USPRM approach. Pathol Res Pract. 2023;241:154273. doi: 10.1016/j.prp.2022.154273. 36563558

[17] Sutaria RG, Sutaria RB. Subacromial bursitis and impingement, musculoskeletal sports and spine disorders: a comprehensive guide. New York: Springer; 2018. p. 51–54.

[18] Allen GM. The diagnosis and management of shoulder pain. J Ultrason. 2018;18(74):234–239. doi: 10.15557/JoU.2018.0034. 30451406 PMC6442215

[19] Achilova F, Daher M, Nassar JE, et al. Frozen shoulder: diagnosis and treatment of adhesive capsulitis. Am J Med. 2026;139(5):598–605. doi: 10.1016/j.amjmed.2026.01.021. 41581632

[20] Alomari A. Current concepts on the intervention for adhesive capsulitis. Explor Musculoskelet Dis. 2025;3:1007110.

[21] Kelly SM, Brittle N, Allen GM. The value of physical tests for subacromial impingement syndrome: a study of diagnostic accuracy. Clin Rehabil. 2010;24(2):149–158. doi: 10.1177/0269215509346103. 20103576

[22] Desouza C, Jani C. Diagnostic accuracy of Neer’s impingement test for subacromial shoulder impingement. Ir J Med Sci. 2025;194(5):1691–1696. doi: 10.1007/s11845-025-04058-4. 40773000

[23] Tamborrini G, Micheroli R, Ricci V, et al. Advancing high-resolution musculoskeletal ultrasound: a histology- and anatomy-driven approach for enhanced shoulder imaging. Part 2: anterior and lateral shoulder. J Ultrason. 2024;24(99):1–12. doi: 10.15557/jou.2024.0032. PMC11665054 39720468

[24] Pesquer L, Borghol S, Meyer P, et al. Multimodality imaging of subacromial impingement syndrome. Skeletal Radiol. 2018;47(7):923–937. doi: 10.1007/s00256-018-2875-y. 29445933

[25] Al Khayyat SG, Stella SM, Trentanni C, et al. Ultrasound definition of subacromial chronic “fibro-adhesive” bursitis and its treatment via ultrasound guided hydrodilation: a prospective pilot study. J Ultrasound. 2024;27(3):599–604. doi: 10.1007/s40477-024-00894-9. 38703325 PMC11333400

[26] Shah D, Alqahtani M, Khan M. Subacromial and subcoracoid bursitis and impingement. In: Orthopaedic sports medicine: an encyclopedic review of diagnosis, prevention, and management. Springer; 2025. p. 1–23. doi:10.1007/978-3-030-65430-6.

[27] Hirji Z, Hunjun JS, Choudur HN. Imaging of the bursae. J Clin Imaging Sci. 2011;1:22. doi: 10.4103/2156-7514.80374. 21966619 PMC3177464

[28] Vosshenrich J, Bruno M, Cantarelli Rodrigues T, et al. Arthroscopy-validated diagnostic performance of 7-minute five-sequence deep learning super-resolution 3-T shoulder MRI. Radiology. 2025;314(2):e241351. doi: 10.1148/radiol.241351. 39964264

[29] Kalra R, Malik S, Abulencia AE, et al. Calcific subcoracoid bursitis. Appl Radiol. 2025. doi:10.37549/AR-D-25-0112.

[30] Albano D, Coppola A, Gitto S, et al. Imaging of calcific tendinopathy around the shoulder: usual and unusual presentations and common pitfalls. Radiol Med. 2021;126(4):608–619. doi: 10.1007/s11547-020-01300-0. 33151457 PMC8007494

[31] Hsu C, Afifi T, Isaac Z. Shoulder pathology on advanced imaging in asymptomatic non-athlete individuals: a narrative review. Pm R. 2024;16(11):1264–1275. doi: 10.1002/pmrj.13169. 38822702

[32] Rhim HC, Ruiz J, Taseh A, et al. Nonsteroidal anti-inflammatory drug injections versus steroid injections in the management of upper and lower extremity orthopedic conditions: a systematic review with meta-analysis. J Clin Med. 2024;13(4):1132. doi: 10.3390/jcm13041132. 38398445 PMC10889729

[33] Ziradkar R, Best TM, Quintero D, et al. Nonsteroidal anti-inflammatory and corticosteroid injections for shoulder impingement syndrome: a systematic review and meta-analysis. Sports Health. 2023;15(4):579–591. doi: 10.1177/19417381221108726. 35897160 PMC10293554

[34] Buchbinder R, Green S, Youd JM, et al. Corticosteroid injections for shoulder pain. Cochrane Database Syst Rev. 1996;2010(1):CD004016. 10.1002/14651858.CD004016 PMC6464922 12535501

[35] Zhu P, Liao B, Wang Z, et al. Resistance band training after triamcinolone acetonide injection for subacromial bursitis: a randomized clinical trial. J Rehabil Med. 2021;53(1):jrm00140. doi: 10.2340/16501977-2752. 33043381 PMC8772364

[36] Yatish R, B KA, Suvarna AA, et al. Effect of ultrasound-guided subacromial bursa injections with various doses of corticosteroid in subacromial bursitis: a retrospective study. Cureus. 2025;17(8):e89307. doi: 10.7759/cureus.89307. 40766090 PMC12322512

[37] Chae J, Jedlicka L. Subacromial corticosteroid injection for poststroke shoulder pain: an exploratory prospective case series. Arch Phys Med Rehabil. 2009;90(3):501–506. doi: 10.1016/j.apmr.2008.10.011. 19254618 PMC4193293

[38] Hopewell S, Keene DJ, Marian IR, et al. Progressive exercise compared with best practice advice, with or without corticosteroid injection, for the treatment of patients with rotator cuff disorders (GRASP): a multicentre, pragmatic, 2 x 2 factorial, randomised controlled trial. Lancet. 2021;398(10298):416–428. doi: 10.1016/s0140-6736(21)00846-1. 34265255 PMC8343092

[39] Oliva F, Marsilio E, Asparago G, et al. The impact of hyaluronic acid on tendon physiology and its clinical application in tendinopathies. Cells. 2021;10(11):3081. doi: 10.3390/cells10113081. 34831304 PMC8625461

[40] Ko JY, Huang CC, Huang PH, et al. Effects of supplementing extracorporeal shockwave therapy to hyaluronic acid injection among patients with rotator cuff lesions without complete tear: a prospective double-blinded randomized study. Int J Surg. 2024;110(12):7421–7433. doi: 10.1097/js9.0000000000002063. 39172722 PMC11634091

[41] Hsieh LF, Hsu WC, Lin YJ, et al. Addition of intra-articular hyaluronate injection to physical therapy program produces no extra benefits in patients with adhesive capsulitis of the shoulder: a randomized controlled trial. Arch Phys Med Rehabil. 2012;93(6):957–964. doi: 10.1016/j.apmr.2012.01.021. 22502793

[42] Saito S, Furuya T, Kotake S. Therapeutic effects of hyaluronate injections in patients with chronic painful shoulder: a meta-analysis of randomized controlled trials. Arthritis Care Res (Hoboken). 2010;62(7):1009–1018. doi: 10.1002/acr.20174. 20235211

[43] Cheng X, Lu M, He F, et al. Effectiveness of ultrasound-guided subacromial bursa injection of betamethasone combined with hyaluronate in treatment of subacromial bursitis. Chin J Med Ultrasound. 2015;12(06):494.

[44] Nakamura Y, Gotoh M, Mitsui Y, et al. Preoperative hyaluronic acid injection modulates postoperative functional outcome in patients undergoing arthroscopic rotator cuff repair. J Orthop Surg Res. 2020;15(1):204. doi: 10.1186/s13018-020-01715-5. 32493376 PMC7268750

[45] Min KS, St Pierre P, Ryan PM, et al. A double-blind randomized controlled trial comparing the effects of subacromial injection with corticosteroid versus NSAID in patients with shoulder impingement syndrome. J Shoulder Elbow Surg. 2013;22(5):595–601. doi: 10.1016/j.jse.2012.08.026. 23177167

[46] Goyal T, Paul S, Sethy SS, et al. Outcomes of ketorolac versus depomedrol infiltrations for subacromial impingement syndrome: a randomized controlled trial. Musculoskelet Surg. 2022;106(1):29–34. doi: 10.1007/s12306-020-00667-7. 32445077

[47] Kim YB, Lee WS, Won JS. The effects of a single-dose subacromial injection of a nonsteroidal anti-inflammatory drug in geriatric patients with subacromial impingement syndrome: a randomized double-blind study. Clin Shoulder Elb. 2021;24(1):4–8. doi: 10.5397/cise.2021.00052. 33652505 PMC7943380

[48] Aksakal M, Ermutlu C, Özkaya G, et al. Lornoxicam injection is inferior to betamethasone in the treatment of subacromial impingement syndrome: a prospective randomized study of functional outcomes. Orthopade. 2017;46(2):179–185. doi: 10.1007/s00132-016-3302-5. 27468823

[49] Capotosto S, Nazemi AK, Komatsu DE, et al. Prolotherapy in the treatment of sports-related tendinopathies: a systematic review of randomized controlled trials. Orthop J Sports Med. 2024;12(11):23259671241275087. doi: 10.1177/23259671241275087. 39502373 PMC11536850

[50] Catapano M, Zhang K, Mittal N, et al. Effectiveness of dextrose prolotherapy for rotator cuff tendinopathy: a systematic review. Pm R. 2020;12(3):288–300. doi: 10.1002/pmrj.12268. 31642203

[51] Lin LC, Lee YH, Chen YW, et al. Comparison clinical effects of hypertonic dextrose and steroid injections on chronic subacromial bursitis: a double-blind randomized controlled trial. Am J Phys Med Rehabil. 2023;102(10):867–872. doi: 10.1097/phm.0000000000002232. 36897810

[52] Chang YJ, Chang FH, Hou PH, et al. Effects of hyperosmolar dextrose injection in patients with rotator cuff disease and bursitis: a randomized controlled trial. Arch Phys Med Rehabil. 2021;102(2):245–250. doi: 10.1016/j.apmr.2020.08.010. 32926850

[53] Omoigui S, Irene S. Subcutaneous injection of anakinra in patients with shoulder pain due to rotator cuff tendonitis and subacromial bursitis. Pain Med. 2004;5(2):229–230. doi: 10.1111/j.1526-4637.2004.04022.x. 15209987

[54] Carroll MB, Motley SA, Wohlford S, et al. Rilonacept in the treatment of subacromial bursitis: A randomized, non-inferiority, unblinded study versus triamcinolone acetonide. Joint Bone Spine. 2015;82(6):446–450. doi: 10.1016/j.jbspin.2015.02.009. 26184525

[55] Muench LN, Tamburini L, Kriscenski D, et al. The effect of augmenting suture material with magnesium and platelet-rich plasma on the in vitro adhesion and proliferation potential of subacromial bursa-derived progenitor cells. JSES Int. 2023;7(6):2367–2372. doi: 10.1016/j.jseint.2023.06.027. 37969491 PMC10638578

[56] Šmíd P, Hart R, Komzák M, et al. Treatment of the shoulder impingement syndrome with PRP injection. Acta Chir Orthop Traumatol Cech. 2018;85(4):261–265. doi: 10.55095/achot2018/045. 30257756

[57] Pasin T, Ataoğlu S, Pasin Ö, et al. Comparison of the effectiveness of platelet-rich plasma, corticosteroid, and physical therapy in subacromial impingement syndrome. Arch Rheumatol. 2019;34(3):308–316. doi: 10.5606/ArchRheumatol.2019.7225. 31598597 PMC6768781

[58] Say F, Gurler D, Bulbul M. Platelet-rich plasma versus steroid injection for subacromial impingement syndrome. J Orthop Surg. 2016;24(1):62–66. doi: 10.1177/230949901602400115. 27122515

[59] Kang H, Jiang H, Chai DD, et al. Comparison of the efficacy of subacromial injection with sodium bicarbonate versus corticosteroid in patients with chronic subacromial bursitis: a prospective, randomized and controlled study. Int J Clin Exp Med. 2016;9(10):18972–18980.

[60] Lee JH, Lee SH, Song SH. Clinical effectiveness of botulinum toxin type B in the treatment of subacromial bursitis or shoulder impingement syndrome. Clin J Pain. 2011;27(6):523–528. doi: 10.1097/AJP.0b013e31820e1310. 21368663

[61] Ayekoloye CI, Nwangwu O. Ultrasound-guided versus anatomic landmark-guided steroid injection of the subacromial bursa in the management of subacromial impingement: a systematic review of randomised control studies. Indian J Orthop. 2020;54(Suppl 1):10–19. doi: 10.1007/s43465-020-00148-w. 32952904 PMC7474019

[62] Deng X, Zhu S, Li D, et al. Effectiveness of ultrasound-guided versus anatomic landmark-guided corticosteroid injection on pain, physical function, and safety in patients with subacromial impingement syndrome: a systematic review and meta-analysis. Am J Phys Med Rehabil. 2022;101(12):1087–1098. doi: 10.1097/phm.0000000000001940. 34966059 PMC9668382

[63] Salce G, Jačisko J, Ricci V, et al. EURO-MUSCULUS/USPRM phantom recipe for (musculoskeletal) interventional ultrasound training. Eur J Phys Rehabil Med. 2025;61(1):102–108. doi: 10.23736/s1973-9087.24.08643-x. 40008913 PMC12505715

[64] Lähdeoja T, Karjalainen T, Jokihaara J, et al. Subacromial decompression surgery for adults with shoulder pain: a systematic review with meta-analysis. Br J Sports Med. 2020;54(11):665–673. doi: 10.1136/bjsports-2018-100486. 30647053

[65] Vandvik PO, Lähdeoja T, Ardern C, et al. Subacromial decompression surgery for adults with shoulder pain: a clinical practice guideline. BMJ. 2019;364:l294. doi: 10.1136/bmj.l294. 30728120

[66] Lavoie-Gagne O, Farah G, Lu Y, et al. Physical therapy combined with subacromial cortisone injection is a first-line treatment whereas acromioplasty with physical therapy is best if nonoperative interventions fail for the management of subacromial impingement: a systematic review and network meta-analysis. Arthroscopy. 2022;38(8):2511–2524. doi: 10.1016/j.arthro.2022.02.008. 35189304

[67] Page MJ, Green S, McBain B, et al. Manual therapy and exercise for rotator cuff disease. Cochrane Database Syst Rev. 2016;2016(6):Cd012224. doi: 10.1002/14651858.Cd012224. 27283590 PMC8570640

